# Mendelian randomization to explore the direct or mediating associations between socioeconomic status and lung cancer

**DOI:** 10.3389/fonc.2023.1143059

**Published:** 2023-05-03

**Authors:** Hong Wu, Jing Yang, Hui Wang, Lei Li

**Affiliations:** ^1^ Department of Oncology, Binzhou Medical University Hospital, Binzhou, Shandong, China; ^2^ Department of Research, Guangxi Medical University Cancer Hospital, Nanning, Guangxi, China; ^3^ Department of Orthopaedic Trauma and Hand Surgery, The First Affiliated Hospital of Guangxi Medical University, Nanning, Guangxi, China

**Keywords:** lung cancer, socioeconomic, GWAS, SNP, Mendelian randomization

## Abstract

**Objective:**

The purpose of this study was to verify whether there are direct or mediated causal associations between socioeconomic status and lung cancer.

**Methods:**

Pooled statistics were obtained from corresponding genome-wide association studies. The inverse-variance weighted, weighted median, MR−Egger, MR-PRESSO and contamination-mixture methods were used as supplements to Mendelian randomization (MR) statistical analysis. Cochrane’s Q value and the MR−Egger intercept were used for sensitivity analysis.

**Results:**

In the univariate MR analysis, household income and education had protective effects on overall lung cancer (income: *P* = 5.46×10^-4^; education: *P* = 4.79×10^-7^) and squamous cell lung cancer (income: *P* = 2.67×10^-3^; education: *P* = 1.42×10^-10^). Smoking and BMI had adverse effects on overall lung cancer (smoking: *P* = 2.10×10^-7^; BMI: *P* = 5.67×10^-4^) and squamous cell lung cancer (smoking: *P* = 5.02×10^-6^; BMI: *P* = 2.03×10^-7^). Multivariate MR analysis found that smoking and education were independent risk factors for overall lung cancer (smoking: *P* = 1.96×10^-7^; education: *P* = 3.11×10^-3^), while smoking was an independent risk factor for squamous cell lung cancer (*P* = 2.35×10^-6^). Smoking, education, and household income mediate the effect of BMI on overall lung cancer (smoking 50.0%, education 49.2%, income 25.3%) and squamous cell lung cancer (smoking 34.8%, education 30.8%, income 21.2%). Smoking, education, and BMI mediate the effect of income on overall lung cancer (smoking 13.9%, education 54.8%, BMI 9.4%) and squamous cell lung cancer (smoking 12.6%, education 63.3%, BMI 11.6%). Smoking, BMI, and income mediate the effect of education on squamous cell lung cancer (smoking 24.0%, BMI 6.2%, income 19.4%).

**Conclusion:**

Income, education, BMI, and smoking are causally associated with both overall lung cancer and squamous cell lung cancer. Smoking and education are independent association factors for overall lung cancer, while smoking is an independent association factor for squamous cell lung cancer. Smoking and education also play important mediating roles in overall lung cancer and squamous cell lung cancer. No causal relationship was found between multiple risk factors associated with socioeconomic status and lung adenocarcinoma.

## Introduction

Cancer is the leading cause of death in countries around the world (1). According to Global Cancer Statistics 2020, lung cancer is the leading cause of cancer incidence and mortality in men, whereas it has the third highest incidence and the second highest mortality in women. Lung cancer, with a global incidence of 11.4%, ranks second among all new cancer cases. It remains the leading cause of cancer death (18%) (2). Socioeconomic status (SES) has been found to be associated with different types of cancer (3). It is a complex factor that can cover multiple dimensions of an individual’s social and economic life circumstances and can be measured using information about education, income, and/or occupation (4). In addition, one study showed strong associations between SES, as measured by education level, household wealth and occupational rank, and smoking (5). A study of socioeconomic and alcohol consumption found that individuals with lower education levels and living in poverty (low income) were associated with higher levels of alcohol consumption (6). People in middle-income countries drink more alcohol than those in high-income countries (6). A cross-sectional study of a PERSIAN cohort of 20,000 Iranian adults found that the most important variables influencing higher body mass index (BMI) levels are SES (75.8%) and education level (-4.1%) (7). Another cross-sectional study based on data from the 2008 Canadian Community Health Survey found a slight association between occupational physical activity and BMI in women, while no association between occupational physical activity and BMI was detected in men (8). Therefore, SES is directly or indirectly associated with education, household income, smoking, alcohol consumption, BMI, and physical activity. This study will evaluate the causal relationship between SES and lung cancer from these dimensions.

The direct effect of smoking (both active and passive) on the development of lung cancer has been confirmed in many studies (9). Education level was an important independent predictor of lung cancer when appropriate follow-up was conducted for incidental findings of clinically significant lung cancer (10). A study based on population epidemiology in Japan found a strong positive association between alcohol consumption and lung cancer in women, but this association almost disappeared after adjusting for smoking (11). A lower BMI was also found to be associated with an increased risk of lung cancer in men in the Japanese population (12). However, physical activity was not found to reduce the risk of lung cancer (13, 14). Earlier studies have shown an increased risk of lung cancer in low SES groups, and the association may be mediated by unexplained smoking exposure, lifestyle or occupational hazards (15, 16).

This study applied Mendelian randomization (MR) to explore factors potentially associated with SES, including education, household income, smoking, alcohol consumption, BMI, and physical activity, and to assess their association with lung cancer. MR is a data analysis method mainly used in epidemiological etiological inference in recent years. Traditional observational epidemiological studies have encountered many challenges in identifying disease etiology and inferring causality, such as reverse causal associations, potential confounders, and exposure factors with minor effects. MR, which uses genetic variation as instrumental variables (IVs) in the study of exposure factors to infer the association between gene-determined phenotypes and diseases, is not affected by the traditional observational interference factors mentioned above (17). Effective IV selection for MR requires the following three key assumptions. Relevance assumption is a genetic variation associated with risk factors for interest; in independence assumption, there are no unmeasurable confounding factors in the association between genetic variation and outcome; and in exclusion restriction, genetic variation cannot directly influence the outcome except through risk factors (18). In this study, univariate, multivariate and mediating MR methods were used to explore the association of factors related to SES with lung cancer. We further explored the direct or indirect role of risk factors and the proportion of indirect risk factors mediated by other factors.

## Material and methods

### Genome-wide association study summary statistics

Education level has certain heritability. James et al. conducted a large-scale genetic association analysis of educational attainment in a sample of approximately 1.1 million individuals and identified 1,271 independent genome-wide significant single nucleotide polymorphisms (SNPs). The study measured the number of years of schooling completed by the age of at least 30. All association analyses were performed in a sample limited to individuals of European ancestry. The combination of education and the three related cognitive phenotypes explained 11-13% of the differences in educational attainment and 7-10% of the differences in cognitive performance (19).

Summary data on household income came from the consortium of the MRC-integrative epidemiology unit, which Ben Elsworth summarized in 2018. It included a 397,751 European population from the UK Biobank, with 9,851,869 SNPs linked to household income (https://gwas.mrcieu.ac.uk/datasets/ukb-b-7408/). The UK Biobank is a large prospective cohort study of approximately 500,000 adults (40-69 years of age) from 22 centers in the United Kingdom (20).

Liu et al. studied the genetic etiology of tobacco and alcohol use in up to 1.2 million Europeans and identified 566 genetic variants associated with multiple stages of tobacco use as well as alcohol use (21). Phenotypes associated with the initiation of smoking included the age at which regular smoking began and whether regular smoking occurred. Phenotypes associated with the severity of smoking were measured by the number of cigarettes smoked per day. The phenotype of smoking cessation was evaluated for previous smokers. Alcohol-related phenotypes were measured using weekly alcohol consumption (21). The World Health Organization defines a smoker as someone who has smoked continuously or cumulatively for six months or more during their lifetime. Therefore, the long-term behavior of regular smoking is more representative of an individual’s smoking behavior than the age of initiation, the number of cigarettes smoked per day, and cessation. Our study included SNPs associated with regular smoking and weekly alcohol consumption as associated phenotypes for smoking and alcohol consumption, respectively.

Yengo et al. conducted a genome-wide association study (GWAS) of BMI in approximately 250,000 European participants and found approximately 100 independent SNPs. The results were pooled with a GWAS of BMI from the UK Biobank involving approximately 450,000 people of European ancestry. The meta-analysis reached 700,000 people and ultimately identified approximately 941 nearly independent BMI-related SNPs (*P<* 1×10^-8^), which explained approximately 6% of the variance in BMI (22).

Klimentidis et al. identified multiple variants in a GWAS of habitual physical activity in more than 377,000 UK Biobank participants. The phenotypes associated with physical activity were divided into four categories, including moderate physical activity, vigorous physical activity, strenuous sports or other exercise, and accelerometer-based physical activity (23). Strenuous exercise or other exercise-related phenotypes were selected for physical activity exposure in this study. Because it was a review of the previous 4 weeks of physical activity, the assessed individuals spent 2-3 or more days per week in physical sports or other exercise, each time lasting 15-30 minutes or more.

The variants associated with lung cancer phenotypes in this study were obtained from the International Lung Cancer Consortium (ILCCO) using the MRBase database (https://www.mrbase.org/). Summary statistics were obtained from four GWAS meta-analyses of European ancestry: the MD Anderson Cancer Center (MDACC) GWAS, the Institute of Cancer Research (ICR) GWAS, the National Cancer Institute (NCI) GWAS and the International Agency for Research on Cancer (IARC) GWAS (24). A total of 27,209 subjects were included (11,348 cases and 15,861 controls). Of the subtypes of lung cancer, 3,275 cases were defined as squamous cell lung cancer, and 3,442 cases were lung adenocarcinoma. There was no sample overlap between the exposures and outcomes selected for this study.

### Mendelian randomization statistical analysis

In this study, univariate MR analysis was used to verify the causal relationship between multiple factors related to SES and lung cancer. Multivariate MR was used to distinguish whether risk factors were independent after evidence showed that multiple risk factors were associated with lung cancer. The independent risk factor was further analyzed by mediating Mendelian randomization to explore the proportion of it mediated by other risk factors. The proportion of the effect that is mediated by any of the potential mediators was estimated using the following equation (standard error estimated using the error propagation method):


$$\text{E }\left(\%\right)={\frac{\sum_{K=1}^{\text{K}}\beta 1*\beta 2_{k}}{\sum_{K=1}^{\text{K}}\beta 3+\beta 1*\beta 2}}_{k}$$

where the regression coefficient β1 is the MR effect of the independent risk factor on the mediator, β2 is the MR effect of the mediator with lung cancer adjusted for the independent risk factor, and β3 is the MR effect of the independent risk factor on lung cancer adjusted for the potential mediator. All regression coefficients were derived from MR instrumental analysis using the inverse-variance weighted (IVW) method, assuming no correlation between the mediators (25–27).

Genome-wide significant single nucleotide polymorphisms (SNPs) (*P*< 5×10^-8^) associated with SES risk factors (education, household income, smoking, alcohol consumption, BMI, and physical activity) were selected as IVs. SNPs selected as IVs with linkage disequilibrium (r2 = 0.001 and KB=10000) and a minor allele frequency of less than 0.01 were removed. Phenotypic variation explained by SNPs was calculated using the formula: R^2^ = 2 × beta^2^ ×(1-EAF)× EAF/SD^2^ (EAF, effect allele frequency; SD, standard deviation; beta refers to the effect of each SNP on the exposure) (28). The weak instrumental variable bias of SNPs was evaluated by the F statistic ((N − k − 1)/k) × (R^2^/(1 − R^2^); N, the sample size; k, number of SNPs) (29). SNPs with an association strength greater than 10 with exposure phenotypes were included in the study.

IVW was used as the primary analytical method to evaluate the effect between exposure and outcome (30). The weighted median (31), MR−Egger (32), MR-PRESSO (33) and contamination-mixture (34) methods were used as supplements to MR statistical analysis. Cochrane’s Q value (35) and the MR−Egger intercept (32) were used for sensitivity analysis to assess heterogeneity and horizontal pleiotropy. When horizontal pleiotropy existed, MR-PRESSO could remove outliers based on the IVW method to provide an estimation of the causal effect again (33). When neither heterogeneity nor horizontal pleiotropy existed, the IVW method was considered the primary assessment method. When the weighted median and IVW were in the same direction and there was heterogeneity, the results of the weighted median method were accepted. Because the weighted median estimate provides an unbiased effect even if up to 50% of genetic IVs are invalid, IVW requires that all SNPS used as IVs are valid (31). To conclude, for there to be a causal relationship between exposure and outcome, the P value of the results of MR analysis should be less than the significance level of 8.33×10^-3^ corrected by Bonferroni (P value threshold = 0.05/6, corrected for 6 pairs of exposure and outcome).

All MR analyses were performed using the “TwoSampleMR (36)”, “MR-PRESSO (33)” and “MendelianRandomization (37)” packages of R software version 4.1.1.

## Results

The number of SNPs as instrumental variables for the six risk factors associated with SES ranged from 14 to 507. The total explained variance and F statistics of the selected instrumental variables are shown in **Supplementary Table S1**. Genome-wide significant SNPs for the six risk factors associated with SES that were extracted as IVs are characterized in **Supplemental Tables S2**–**7**.

**Figure 1:**
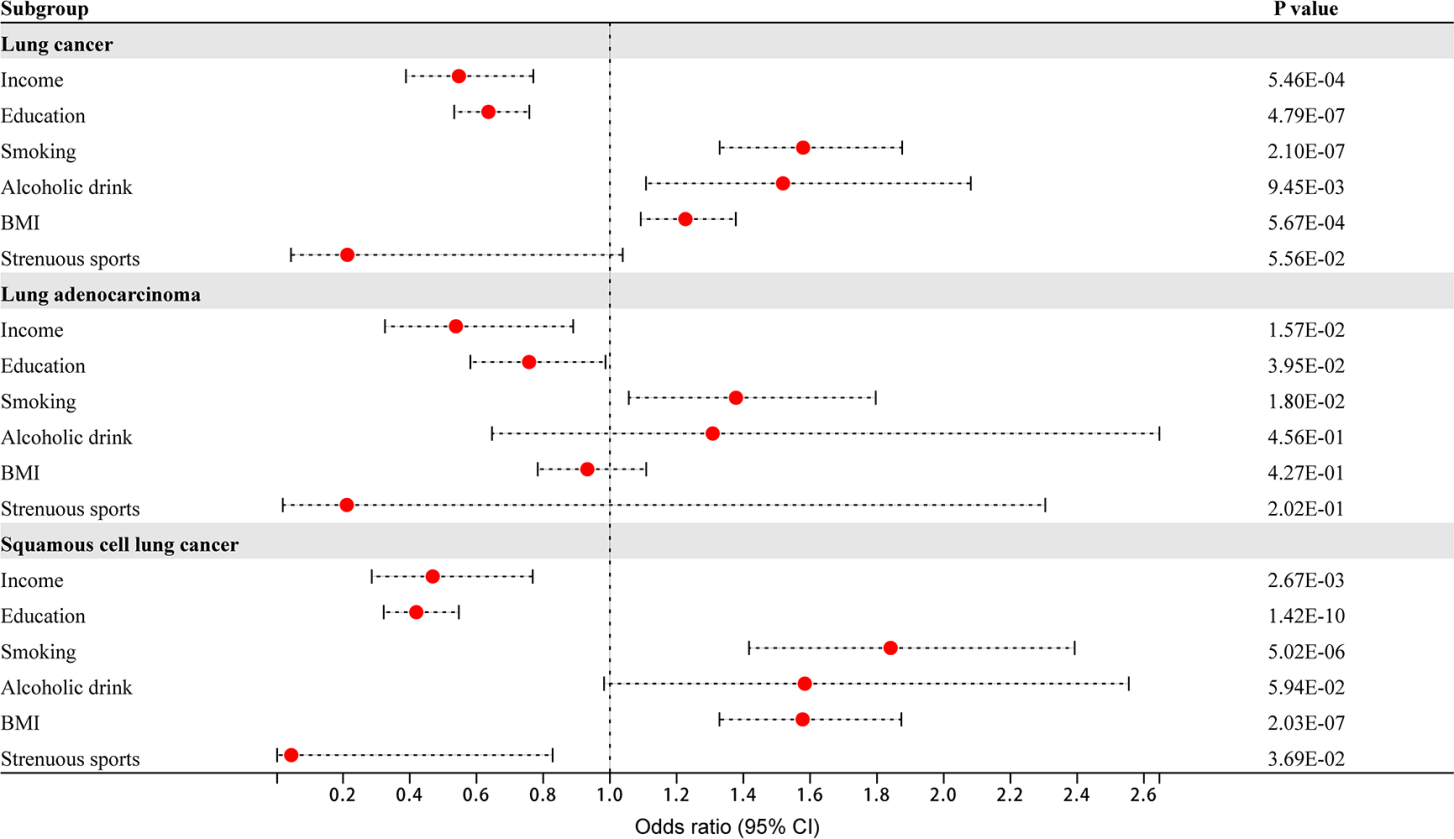
The effect of 6 dimensional factors of socioeconomic status on the risk of lung cancer and its subtypes.

In the univariate MR analysis, lung cancer as the outcome was divided into three categories: lung cancer (including adenocarcinoma and squamous cell carcinoma), lung adenocarcinoma, and squamous cell lung cancer. There was evidence that household income and education had protective effects on overall lung cancer in the main IVW method (income: OR 0.55, 95% CI 0.39–0.77, *P* = 5.46×10^-4^; education: OR 0.64, 95% CI 0.53–0.76, *P* = 4.79×10^-7^). Smoking and BMI had adverse effects on overall lung cancer (smoking: OR 1.58, 95% CI 1.33–1.88, *P* = 2.10×10^-7^; BMI: OR 1.23, 95% CI 1.09–1.38, *P* = 5.67×10^-4^). There was no significant association between alcohol consumption, strenuous sports and overall lung cancer (alcohol drink: OR 1.52, 95% CI 1.11–2.08, *P* = 9.45×10^-3^; strenuous sports: OR 0.21, 95% CI 0.04–1.04, *P* = 5.56×10^-2^; *P* value< 8.33×10^-3^ corrected by Bonferroni) (**Figure 1**). Income, education, smoking, alcohol consumption, BMI and strenuous sports were not significantly associated with lung adenocarcinoma (income: OR 0.54, 95% CI 0.33–0.89, *P* = 1.57×10^-2^; education: OR 0.76, 95% CI 0.58–0.99, *P* = 3.95×10^-2^; smoking: OR 1.38, 95% CI 1.06–1.80, *P* = 1.80×10^-2^; alcohol drink: OR 1.31, 95% CI 0.65–2.65, *P* = 4.56×10^-1^; BMI: OR 0.93, 95% CI 0.78–1.11, *P* = 4.27×10^-1^; strenuous sports: OR 0.21, 95% CI 0.02–2.30, *P* = 2.02×10^-1^; *P* value< 8.33×10^-3^ corrected by Bonferroni) (**Figure 1**). There was evidence that household income and education had protective effects against squamous cell lung cancer (income: OR 0.47, 95% CI 0.29–0.77, *P* = 2.67×10^-3^; education: OR 0.42, 95% CI 0.32–0.55, *P* = 1.42×10^-10^). Smoking and BMI had adverse effects on squamous cell lung cancer (smoking: OR 1.84, 95% CI 1.42–2.39, *P* = 5.02×10^-6^; BMI: OR 1.58, 95% CI 1.33–1.87, *P* = 2.03×10^-7^). There was no significant association between alcohol consumption, strenuous sports and squamous cell lung cancer (alcohol drink: OR 1.58, 95% CI 0.98–2.56, *P* = 5.94×10^-2^; strenuous sports: OR 0.04, 95% CI 0.002–0.83, *P* = 3.69×10^-2^; *P* value< 8.33×10^-3^ corrected by Bonferroni) (**Figure 1**). Heterogeneity and horizontal pleiotropy were not observed in the Mendelian randomized sensitivity analysis.

The results of the IVW, weighted median, MR−Egger, MR-PRESSO and contamination-mixture methods for the three types of lung cancer, as well as the sensitivity analysis results of Cochran’s Q and the MR−Egger intercept, are shown in **Supplemental Tables S8**–**10**.

**Figure 2:**
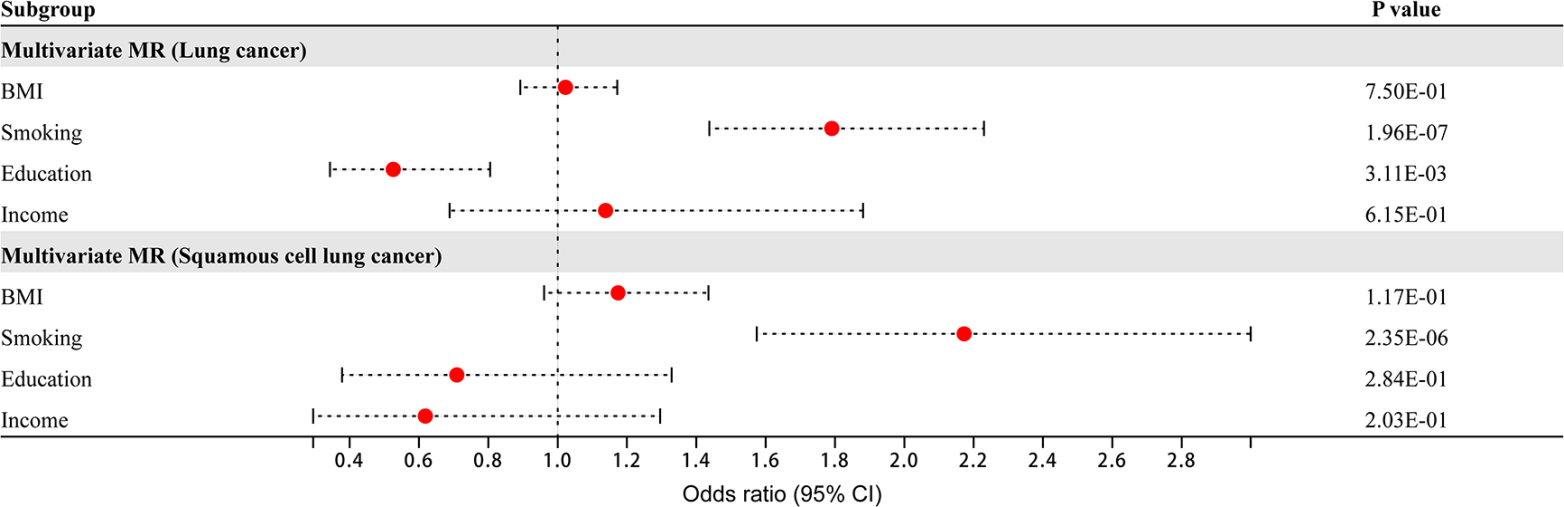
Multivariate Mendelian randomization analysis of overall lung cancer and squamous cell lung cancer.

Four risk factors - BMI, smoking, education, and income - had causal relationships with overall lung cancer and squamous cell lung cancer. Multivariate MR analysis found that smoking and education were independent risk factors for overall lung cancer (smoking: OR 1.79, 95% CI 1.44–2.23, *P* = 1.96×10^-7^; education: OR 0.53, 95% CI 0.34–0.81, *P* = 3.11×10^-3^), while smoking was an independent risk factor for squamous cell lung cancer (OR 2.17, 95% CI 1.57–3.00, *P* = 2.35×10^-6^) (**Figure 2**). BMI and income were nonindependent risk factors for overall lung cancer, while BMI, education and income were nonindependent risk factors for squamous cell lung cancer. These nonindependent risk factors were mediated by other risk factors.

**Figure 3:**
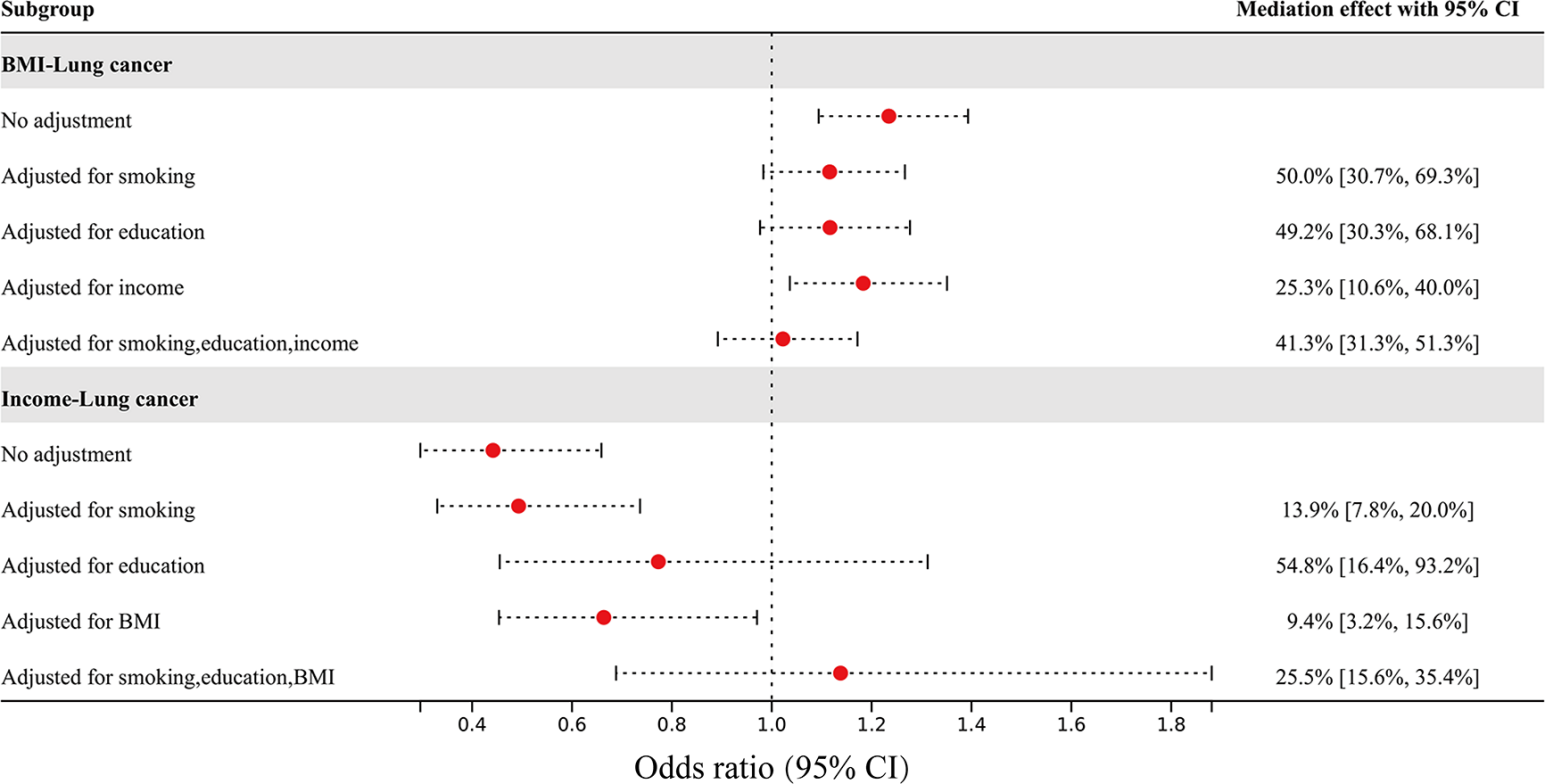
The effect of BMI and income on overall lung cancer risk after adjusting for mediators, and the percentage of mediating factors.

Mediating MR was used to assess the mediating effect of nonindependent risk factors on overall lung cancer through other risk factors, the results of which are shown in **Figure 3**. The effect of BMI on overall lung cancer risk was attenuated from an OR of 1.23 (95% CI 1.09–1.39) to an OR of 1.12 (95% CI 0.98–1.27) after adjusting for smoking, to an OR of 1.12 (95% CI 0.98–1.28) after adjusting for education, to an OR of 1.18 (95% CI 1.04–1.35) after adjusting for income, and to an OR of 1.02 (95% CI 0.89–1.17) after adjusting for all three factors. The mediating percentages of different risk factors between BMI and overall lung cancer risk were smoking at 50.0% (95% CI 30.7-69.3%), education at 49.2% (95% CI 30.3-68.1%), income at 25.3% (95% CI 10.6-40.0%), and all three factors at 41.3% (95% CI 31.3-51.3%). The effect of income on overall lung cancer risk was attenuated from an OR of 0.44 (95% CI 0.30–0.66) to an OR of 0.49 (95% CI 0.33–0.74) after adjusting for smoking, to an OR of 0.77 (95% CI 0.46–1.31) after adjusting for education, to an OR of 0.66 (95% CI 0.45–0.97) after adjusting for BMI, and to an OR of 1.14 (95% CI 0.69-1.88) after adjusting for all three factors. The mediating percentages of different risk factors between household income and overall lung cancer risk were as follows: smoking at 13.9% (95% CI 7.8-20.0%), education at 54.8% (95% CI 16.4-93.2%), BMI at 9.4% (95% CI 3.2-5.6%), and all three factors at 25.5% (95% CI 15.6-35.4%).

**Figure 4:**
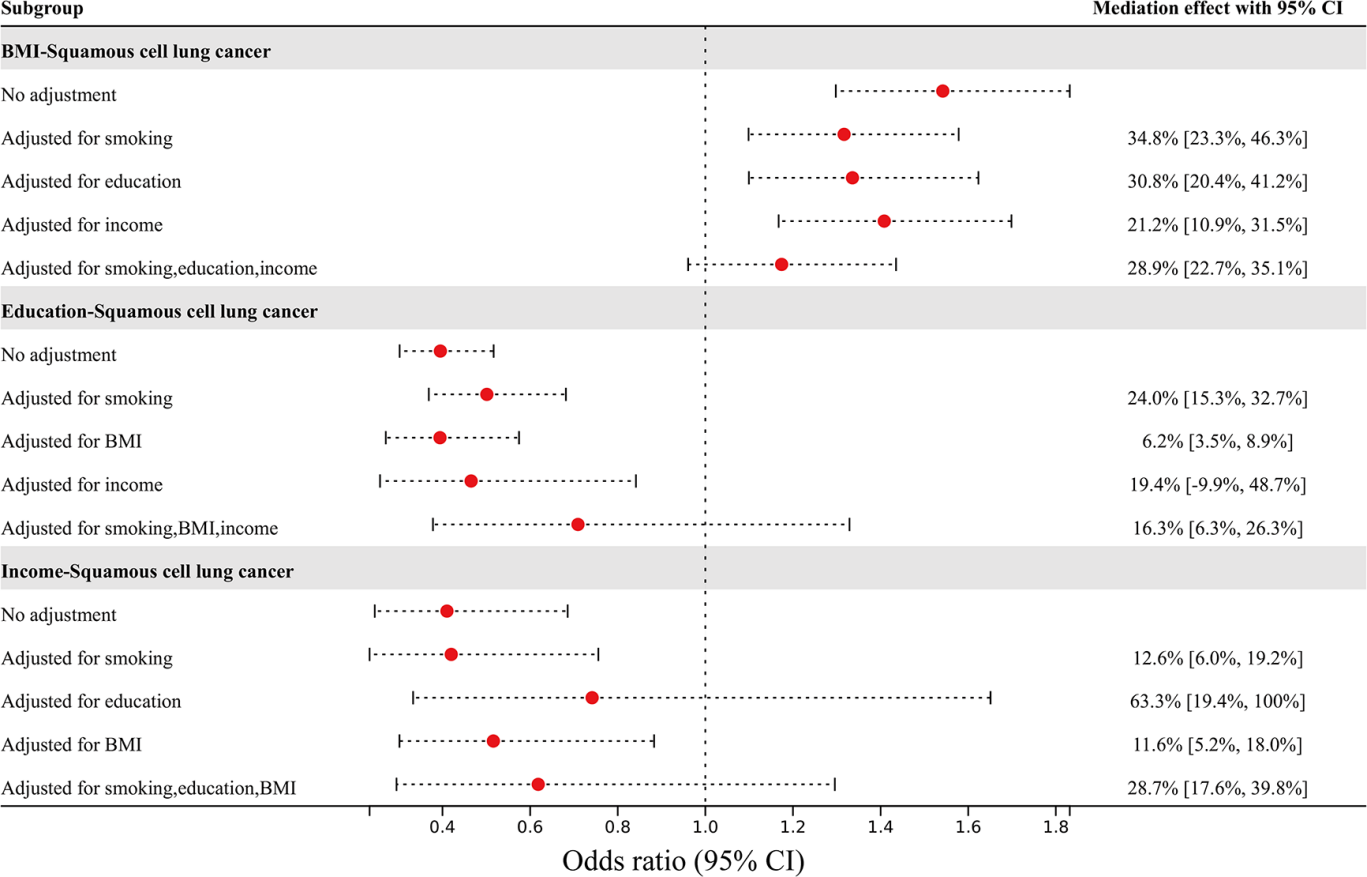
The effects of BMI, education, and income on squamous cell lung cancer risk after adjusting for mediators, and the percentage of mediating factors.

The mediating effect of nonindependent risk factors on squamous cell lung cancer through other risk factors is shown in **Figure 4**. The effect of BMI on squamous cell lung cancer risk was attenuated from an OR of 1.54 (95% CI 1.30–1.83) to an OR of 1.32 (95% CI 1.10–1.58) after adjusting for smoking, to an OR of 1.34 (95% CI 1.10–1.62) after adjusting for education, to an OR of 1.41 (95% CI 1.17–1.70) after adjusting for income, and to an OR of 1.17 (95% CI 0.96–1.44) after adjusting for all three factors. The mediating percentages of different risk factors between BMI and squamous cell lung cancer risk were as follows: smoking at 34.8% (95% CI 23.3-46.3%), education at 30.8% (95% CI 20.4-41.2%), income at 21.2% (95% CI 10.9-31.5%), and all three factors at 28.9% (95% CI 22.7-35.1%). The effect of education on squamous cell lung cancer risk was attenuated from an OR of 0.40 (95% CI 0.30–0.52) to an OR of 0.50 (95% CI 0.37–0.68) after adjusting for smoking, to an OR of 0.39 (95% CI 0.27–0.57) after adjusting for BMI, to an OR of 0.47 (95% CI 0.26–0.84) after adjusting for income, and to an OR of 0.71 (95% CI 0.38–1.33) after adjusting for all three factors. The mediating percentages of different risk factors between education and squamous cell lung cancer risk were as follows: smoking at 24.0% (95% CI 15.3-32.7%), BMI at 6.2% (95% CI 3.5-8.9%), income at 19.4% (95% CI -9.9-48.7%), and all three factors at 16.3% (95% CI 6.3-26.3%). The effect of income on squamous cell lung cancer risk was attenuated from an OR of 0.41 (95% CI 0.25–0.69) to an OR of 0.42 (95% CI 0.23–0.76) after adjusting for smoking, to an OR of 0.74 (95% CI 0.33–1.65) after adjusting for education, to an OR of 0.52 (95% CI 0.30–0.88) after adjusting for BMI, and to an OR of 0.62 (95% CI 0.30–1.30) after adjusting for all three factors. The mediating percentages of different risk factors between income and squamous cell lung cancer risk were smoking at 12.6% (95% CI 6.0-19.2%), education at 63.3% (95% CI 19.4-100%), BMI at 11.6% (95% CI 5.2-18.0%), and all three factors at 28.7% (95% CI 17.6-39.8%). The data used in the mediated MR analysis for multiple independent risk factors are shown in **Supplemental Tables S11**–**13**.

## Discussion

In this study, we found that household income, education, smoking, and BMI related to SES were associated with both overall lung cancer and squamous cell lung cancer. There was no evidence of a causal relationship between the factors selected as SES and lung adenocarcinoma. Smoking and education were independently associated with overall lung cancer, while smoking was an independent risk factor for squamous cell lung cancer.

Consistent with our findings, education has been found to be associated with lung cancer in multiple studies. The 2021 National Health Survey of the United States found that lung cancer patients aged 45 or older had the highest number of education degrees below high school (8.6%). The rate of lung cancer decreased with increasing education level, with the lowest prevalence among those with a bachelor’s degree or higher (1.6%) (38). Another cohort study of Norwegians also found that low education increased the risk of dying from lung cancer (39). A Mendelian randomized study on education and lung cancer also showed that low education was a causal risk factor in the development of lung cancer (40). There is much research evidence on the causal relationship between smoking and lung cancer. Smoking is an important risk factor for lung cancer and is independently associated with a higher risk of lung cancer (41, 42). Smoking cessation can improve lung cancer incidence and survival rates, and even those who quit in middle age can avoid most of the risk of lung cancer (43–46). An observational trial of the mediating role of smoking in the relationship between education and lung cancer found that the indirect effects of smoking varied by level of education, with the strongest effects observed for those with the least education (47). Therefore, in this study, we also demonstrated causal relationships between smoking, education and overall lung cancer. However, neither smoking nor education was an independent risk factor for squamous cell lung cancer subtypes.

Associations of BMI with lung cancer have been reported across ethnic groups and across observational cohort studies; however, their findings are ambiguous. Based on relevant studies of the International Lung Cancer Consortium and the Chinese population, it was found that both an increase and a decrease in BMI could increase the risk of lung cancer and reduce the survival rate of lung cancer (48, 49). Decreased BMI was also found to be associated with poorer overall survival of lung cancer, while weight gain increased the risk of death but was not statistically significant (50). Other studies have found a positive correlation between BMI and the incidence of lung cancer or lung adenocarcinoma (51, 52). Additionally, an observational study showed that higher BMI was associated with a lower risk of lung cancer (53). From this, we can see that the results of observational studies vary and that racial differences may be one reason for this difference (54, 55). We included people of European descent in this study and concluded that an increase in BMI increases the risk of both overall lung cancer and squamous cell lung cancer. However, the causal relationship between BMI and lung cancer was not an independent risk factor; it was mediated by smoking, education and income, with smoking having the strongest mediating effect, followed by education. Another MR study also found a mediating role of smoking in the association between BMI and lung cancer (56).

Overall survival of lung cancer was positively correlated with high income of patients and was not correlated with race (57). A lower economic gradient was associated with higher mortality from lung cancer (58). Similarly, studies found that the prevalence and mortality of lung cancer were higher among people with economic poverty and low education levels (59, 60). In this study, household income was negatively correlated with overall lung cancer and squamous cell lung cancer, but it was not an independent association factor. Education, smoking and BMI play a mediating role between the two, with education playing the strongest mediating role, followed by smoking. The higher the level of education, the lower the overall risk of lung cancer. Education was an independent association factor for overall lung cancer but not squamous cell lung cancer. The negative correlation between education and squamous cell lung cancer was mediated by smoking, income and BMI, among which smoking was the most important mediator.

Mendelian randomization has some limitations that need to be noted. First, exposures and outcomes can only be selected based on the limited GWAS data available, which has certain limitations. In addition, estimates of intermediary proportions may be biased due to the noncollapsibility of ORs (27). Finally, the study was based only on populations of European descent. This conclusion only applies to European ancestry and cannot represent all ethnic groups. Further verification is needed for other ancestry groups.

## Conclusion

Income, education, BMI, and smoking are causally associated with both overall lung cancer and squamous cell lung cancer. Smoking and education are independent association factors for overall lung cancer, while smoking is an independent association factor for squamous cell lung cancer. Second, smoking and education also play important mediating roles in lung cancer: the causal association between BMI and overall lung cancer and squamous cell lung cancer is mainly mediated by smoking and education. The causal association between household income and overall lung cancer and squamous cell lung cancer is mainly mediated by education; finally, the causal association between education and squamous cell lung cancer is mainly mediated by smoking. No causal relationship is found between multiple risk factors associated with socioeconomic status and lung adenocarcinoma.

## Data availability statement

The datasets presented in this study can be found in online repositories. The names of the repository/repositories and accession number(s) can be found in the article/**Supplementary Material.**

## Author contributions

HWu and JY drafted the manuscript and contributed to the conception of the study. HWa helped perform the analysis with constructive discussions. LL gave critical feedback and approved the final version. All authors contributed to the article and approved the submitted version.
